# Development of Patient-Derived Conditionally Reprogrammed 3D Breast Cancer Culture Models for Drug Sensitivity Evaluation

**DOI:** 10.32604/or.2025.069902

**Published:** 2025-12-30

**Authors:** Jing Cai, Haoyun Zhu, Weiling Guo, Ting Huang, Pangzhou Chen, Wen Zhou, Ziyun Guan

**Affiliations:** 1 Research Institute of Medicine, The Sixth Affiliated Hospital, School of Medicine, South China University of Technology, Foshan, 528000, China; 2 Department of Breast Surgery, The Sixth Affiliated Hospital, School of Medicine, South China University of Technology, Foshan, 528000, China; 3 Department of Emergency, The Sixth Affiliated Hospital, School of Medicine, South China University of Technology, Foshan, 528000, China

**Keywords:** Patient-derived breast cancer cells, conditional reprogramming, hydrogel microsphere, 3D culture model, drug screening

## Abstract

**Background:**

Therapeutic responses of breast cancer vary among patients and lead to drug resistance and recurrence due to the heterogeneity. Current preclinical models, however, are inadequate for predicting individual patient responses towards different drugs. This study aimed to investigate the patient-derived breast cancer culture models for drug sensitivity evaluations.

**Methods:**

Tumor and adjacent tissues from female breast cancer patients were collected during surgery. Patient-derived breast cancer cells were cultured using the conditional reprogramming technique to establish 2D models. The obtained patient-derived conditional reprogramming breast cancer (CRBC) cells were subsequently embedded in alginate-gelatin methacryloyl hydrogel microspheres to form 3D culture models. Comparisons between 2D and 3D models were made using immunohistochemistry (tumor markers), MTS assays (cell viability), flow cytometry (apoptosis), transwell assays (migration), and Western blotting (protein expression). Drug sensitivity tests were conducted to evaluate patient-specific responses to anti-cancer agents.

**Results:**

2D and 3D culture models were successfully established using samples from eight patients. The 3D models retained histological and marker characteristics of the original tumors. Compared to 2D cultures, 3D models exhibited increased apoptosis, enhanced drug resistance, elevated stem cell marker expression, and greater migration ability—features more reflective of *in vivo* tumor behavior.

**Conclusion:**

Patient-derived 3D CRBC models effectively mimic the *in vivo* tumor microenvironment and demonstrate stronger resistance to anti-cancer drugs than 2D models. These hydrogel-based models offer a cost-effective and clinically relevant platform for drug screening and personalized breast cancer treatment.

## 1 Introduction

Breast cancer is the second most commonly diagnosed cancer worldwide and the leading cause of cancer-related death among women, with approximately 2.3 million new cases annually [1,2]. It is a global health concern characterized by various molecular subtypes, including Luminal A, Luminal B, human epidermal growth factor receptor 2 (HER-2), and triple-negative breast cancer (TNBC) [3]. Treatment strategies are largely guided by hormone receptor status, especially estrogen receptor (ER), progesterone receptor (PR), and HER-2 [4]. For example, tamoxifen is used for hormone receptor-positive cases [5], while HER2-positive patients are treated with herceptin and docetaxel [6]. TNBC is typically managed with anthracyclines and taxanes [7]. Despite advances in surgery, endocrine therapy, and chemotherapy, both intrinsic and acquired drug resistance remain significant barriers to successful treatment [8–10]. This is largely due to the inherent heterogeneity of breast tissue, which becomes even more pronounced in tumor states [11,12]. Molecular classification alone is often insufficient to capture the complexity of individual tumors or to predict therapeutic outcomes [13]. In particular, variations in tumor cell biology and the tumor microenvironment contribute to the wide range of drug responses observed clinically [14,15]. Given these challenges, there is an urgent need for personalized treatment evaluation systems that can account for tumor heterogeneity and provide tailored therapeutic guidance for individual patients.

An effective personalized treatment evaluation system requires overcoming several challenges—foremost among them is the ability to culture patient-derived tumor cells *in vitro*. Cells obtained directly from patient tissues typically exhibit limited proliferation, making it difficult to establish reliable *in vitro* models essential for drug screening. Conditional reprogramming technology addresses this limitation by co-culturing human tumor cells with irradiated mouse fibroblasts in the presence of rho-associated coiled-coil-containing protein kinase (ROCK) inhibitors such as Y-27632. This method supports sustained cell growth while maintaining genomic stability and differentiation potential [16,17]. Conditional reprogramming-derived cells also preserve a degree of tumor heterogeneity. Upon removal of the ROCK inhibitor and feeder cells, the reprogrammed cells can revert to differentiated states, further demonstrating their capacity to reflect the parent tumor’s complexity. As such, conditional reprogramming-based models retain key genetic and phenotypic traits of the original tumors, making them suitable for drug sensitivity testing, molecular biology research, and studies on tumor heterogeneity [18,19]. However, most drug screening still relies on two-dimensional (2D) monolayer cultures, which fail to accurately replicate *in vivo* conditions. In 2D systems, cells exhibit unnatural morphology, lack interactions with the extracellular matrix, and display altered proliferation and differentiation patterns. Although patient-derived xenograft models better simulate the tumor microenvironment, their high cost and complexity limit widespread application for screening purposes. In recent years, three-dimensional (3D) organoid cultures have emerged as effective models for maintaining native cell morphology, proliferation, and intercellular interactions [20,21]. These systems support the stable growth of patient-derived cells and have shown great potential for personalized drug screening platforms [22]. While Matrigel is commonly used to provide structural support in 3D organoid cultures, its high cost and limited long-term stability hinder its broader application. Hydrogels offer a promising alternative by providing a supportive matrix that mimics the extracellular environment. Among them, alginate, a naturally occurring anionic polymer, is favored for its biocompatibility and degradability in 3D culture applications [23]. Gelatin, derived from collagen hydrolysis, enhances cell attachment, while its derivative, gelatin methacryloyl (GelMA), contains methacrylamide groups that closely resemble the native extracellular matrix. GelMA is widely used in biomedical research due to its moldability, stability, and compatibility with living cells [24]. Despite these advantages, the application of hydrogel-based 3D models loaded with patient-derived breast cancer cells for personalized drug screening remains limited in current research.

**Figure 1:**
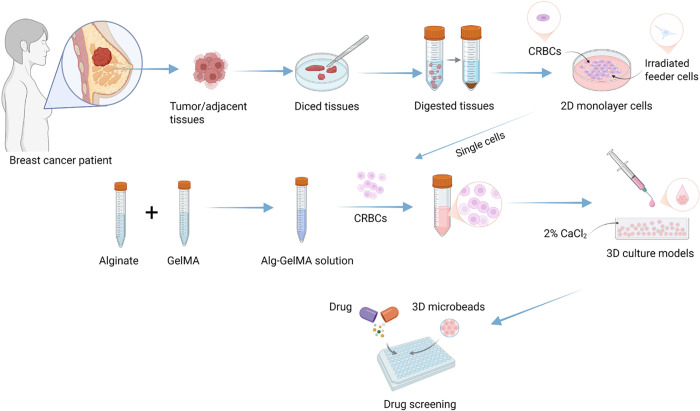
Schematic diagram of experimental design for establishing patient-derived 3D cell culture models and the comprehensive evaluation of the drug sensitivity. Image created with BioRender.com, with permission

This study aims to discuss the function of 3D organoid culture models in conducting clinical precise and personalized drug screening in breast cancer. We developed a patient-derived 3D conditionally reprogrammed breast cancer (CRBC) cell culture models to evaluate the efficacy of various anti-cancer drugs (Fig. 1). We conducted and compared the functions of 2D monolayer cultures and 3D culture models. Our results indicated that, compared to 2D cultures, these CRBC-based 3D models supported stable cell proliferation, better mimicked *in vivo* tumor characteristics, enhanced expression of stemness-related genes, greater migratory capacity and efficacy of various anti-cancer drugs. Hence, our developed patient-derived 3D CRBC culture system potentially serves as a robust platform for personalized drug screening and precision oncology.

## 2 Materials and Methods

### 2.1 Materials

High-glucose Dulbecco’s modified Eagle medium (DMEM), F-12 basic medium, fetal bovine serum (FBS), L-Glutamine, penicillin-streptomycin, and newborn calf serum (NBCS) were purchased from Thermo Fisher Scientific (Waltham, MA, USA). Sodium alginate (Alg, A0682), gelatin methacryloyl (GelMA, EFL-GM-60), amphotericin B (V900919), gentamicin (G1914), human insulin (I264), and cholera toxin (C8052) were obtained from Sigma-Aldrich (Livonia, MI, USA). Calcium chloride (C108383) was from Shanghai Aladdin Biochemical Technology (Shanghai, China), while collagenase IV (0219511090) and DNase I (ICN10057505) were from MP Biomedicals (Santa Ana, CA, USA). The MTS assay kit (G3580) was purchased from Promega (Madison, WI, USA), and the Calcein-AM/PI double-staining kit (C2015M) was from Beyotime Biotech Inc. (Shanghai, China). The ROCK inhibitor Y27632 (10 μM) were purchased from Selleck (Houston, TX, USA). Anti-cancer drugs, including carboplatin (HY-17393), cisplatin (HY-17394), paclitaxel (HY-B0015), toremifene citrate (HY-B0005), docetaxel (HY-B0011), gemcitabine (HY-17026), vinorelbine ditartrate (HY-12053A), epirubicin hydrochloride (HY-13624A), and doxorubicin hydrochloride (HY-15142), were purchased from MedChemExpress (Monmouth Junction, NJ, USA). Their working concentration was shown in Table A2 (Appendix A). All drugs were prepared according to the manufacturer’s instructions and stored at −80°C. Epidermal growth factor (EGF, AF-100-15) and hydrocortisone (G8450) were purchased from PeproTech (Cranbury, NJ, USA) and Solarbio (Beijing, China), respectively. The primary antibodies against GAPDH (AF0006) and β-Actin (AF0003) were purchased from Beyotime (Shanghai, China), and used at 1:2000 dilution. The antibodies against NANOG (F0290), ALDH1A1 (F1023), and SOX-2 (F0310) were purchased from Selleck (Houston, TX, USA) and used at 1:1000 dilution. The goat anti-mouse antibody and goat anti-rabbit antibody were purchased from Cell Signaling Technology (Boston, MA, USA). Primary tissue storage solution (PTSS) was obtained from BioGenous (Suzhou, China). All cell lines were confirmed to be mycoplasma-free by the Mycoalert detection kit (LT07-218, Lonza, Switzerland).

### 2.2 Collection of Patient-Derived Breast Tumors and Adjacent Tissues

Breast cancer and adjacent normal tissues (eight patients) were collected from patients undergoing surgical resection at the Sixth Affiliated Hospital of South China University of Technology. The study was reviewed and approved by the Hospital’s Ethics Committee (No. [2021]-109), and written informed consent was obtained from all participants. Immediately after resection, tumor tissues were separated from adipose tissue, placed in PTSS, and processed within four hours. Clinical data were retrieved from hospital records and are summarized in Table A1 (Appendix A). Each sample was divided into three portions for primary breast cancer cell isolation, histological analysis, and construction of CRBC-derived hydrogel microspheres.

### 2.3 Digestion and Culture of Patient-Derived Breast Tumor Cells

Fresh tumor and normal tissues were rinsed with 95% ethanol, followed by cold PBS (0.01 M, pH 7.4). Tissues were minced into 1–3 mm^3^ pieces and digested in a solution containing 1 mg/mL collagenase IV and 2000 PU/mL DNase I (mixed at a 1:3 ratio) for 6 h at 37°C. Mechanical dissociation was applied post-digestion to release cells. The resulting cells were co-cultured with freshly irradiated Swiss-3T3-J2 mouse fibroblasts (CRL-1658, ATCC, Manassas, VA, USA) in culture medium supplemented with Y27632 at 37°C and 5% CO_2_ for several days. The cells used in this study have been identified by short tandem repeat (STR) and tested for mycoplasma contamination. Fresh medium and fibroblasts were replaced as needed until the primary patient-derived breast cancer cells exhibited extensive proliferation.

### 2.4 Construction and Characteristics of CRBC-Alginate-GelMA (CRBC-Alg-GelMA) Microbeads

Sodium alginate and GelMA were mixed at room temperature in a specific ratio (4:1) to prepare the Alg-GelMA solution. Cultured CRBC cells were digested with 0.25% trypsin and collected after centrifugation (800 g, 5 min). Then, the Alg-GelMA solution was combined with the centrifuged CRBC cells precipitate. The mixture was then dropped into 2% CaCl_2_ solution in a cell culture medium to form CRBC-Alg-GelMA hydrogel microbeads. After standing for 30 s, the beads were exposed to UV light (OmniCure S2000, Excelitas, Pittsburgh, PA, USA) for 30 s. The CaCl_2_ solution was removed, and the microbeads were washed with sterile ultrapure water. The resulting microbead’s diameter was measured using a vernier caliper. Following cultivation in medium at 37°C and 5% CO_2_ for a few days, microbeads were freeze-dried and examined using a field emission scanning electron microscope (JEOLJSM-6700F, JEOL Ltd., Tokyo, Japan) to observe surface morphology.

### 2.5 Dissociation of CRBC-Alg-GelMA Microbeads

After several days of incubation, CRBC-Alg-GelMA microbeads were collected and washed three times with PBS (0.01 M, pH 7.4). The beads were then incubated in a lysis solution composed of 55 nM sodium citrate and complete cell medium mixed 1:1 on a shaker (130 r/min, 10 min, ZHWY-103B, Zhicheng, Shanghai, China) at 37°C. The lysate was monitored under a microscope (BX51, Olympus, Tokyo, Japan) every 15 min. Once the microbeads were fully dissolved, free cells were collected by centrifugation (5804r, Eppendorf, Hamburg, Germany) at 800 rpm for 5 min. These cells were then ready for culture and further analysis.

### 2.6 Histological Staining

Fresh breast cancer tissues were fixed in 4% paraformaldehyde and embedded in paraffin. Sections were cut into 5 μM thickness for further processing. CRBC-Alg-GelMA microbeads were dissociated using 0.3 mg/mL GelMA lysis buffer (EFL-GM-LS-001, Engineering for Life Co., Ltd., Shanghai, China) in a constant temperature shaker (ZHWY-103B, Zhicheng, Shanghai, China) at 37°C. Cells were collected by centrifugation at 800 rpm for 5 min. A total of 1 × 10^6^ CRBC cells were processed into cell blocks using a cell block preparation kit (7401150, Thermo, Waltham, MA, USA) and sectioned. These sections were incubated with 3% H_2_O_2_ for 10 min, blocked for 20 min, and stained overnight at 4°C with primary antibodies against Ki-67 (CST, 9449S, 1:500), anti-ER (Abcam, ab16660, 1:200), anti-PR (Abcam, ab101688, 1:400), and anti-HER2 (CST, 2165S, 1:200), respectively. After incubation with these primary antibodies, sections were treated with a 1:200 diluted secondary antibody for 20 min, followed by incubation with ABC reagent for 30 min using the Vectastain ABC HRP kit (PK-4000, Vector Labs, CA, USA). Each step was followed by three TBS washes per the manufacturer’s protocols. The stained signals were visualized by incubation with DAB reagent kit (K3468, Agilent, Santa Clara, CA, USA) for 30 s. The sections were counterstained with hematoxylin, dehydrated, and mounted. Images were captured using a Nikon ECLIPSE Ci microscope (Nikon, Tokyo, Japan).

### 2.7 Assessment of CRBC Proliferation in 2D and 3D Cultures

The proliferation of patient-derived CRBC cells cultivated in 2D and 3D models was evaluated using MTS assays at various time points. For 2D culture, 3000 cells were seeded per well in 96-well plates. Once cells adhered, 10 μL of MTS reagent and 90 μL of fresh medium were added to each well. After 2 h of incubation at 37°C, optical density (OD) was measured at 490 nm using a microplate reader (Synergy HTX, BioTek, Winooski, VT, USA). For 3D culture, each cell-containing microbead was placed into a well of a 96-well plate with 10 μL of MTS reagent and 90 μL of fresh medium. OD values were measured after 4 h of incubation at 37°C. Cell viability was calculated as the ratio of the OD values of experimental groups to those of the control group.

### 2.8 Live/Dead Cell Staining

Cell viability in both 2D cultures and CRBC-Alg-GelMA microbeads was assessed using a Calcein-AM/Propidium Iodide (PI) assay kit (C2015M, Beyotime, Shanghai, China). Microbeads cultured for 1 and 7 days were selected for analysis. The Calcein-AM and PI reagents were each diluted 1:1000 in the provided dilution buffer. Microbeads were incubated with diluted Calcein-AM for 35 min, followed by diluted PI for 25 min. Live (Calcein-AM positive) and dead (PI positive) cells were visualized using fluorescence microscopy (λex = 497 nm, λem = 518 nm; Nikon Corporation, Tokyo, Japan) and confocal laser scanning microscope (λex = 497 nm, λem = 518 nm; FV3000, Olympus, Tokyo, Japan). Images were captured as z-stacks and combined into composite images using ImageJ version 1.53q (NIH, Bethesda, MD, USA) for analysis.

### 2.9 Flow Cytometry Analysis of CRBC Apoptosis

Apoptosis of CRBC cells in 2D culture and CRBC-Alg-GelMA microbeads were assessed using flow cytometry. Microbeads were collected after several days of culture and dissociated into single cells using the GelMA lysis protocol. Cells were washed with cold PBS (0.01 M, pH 7.4) and centrifuged at 1000 rpm for 3 min. Calcein-AM and PI reagents were diluted 1:1000 in the provided dilution buffer. Cell suspensions were divided into four groups: blank, PI-only, Calcein-AM-only, and Calcein-AM/PI double staining. Flow cytometry was performed using a FACSCanto II system (BD Biosciences, Mississauga, CA, USA) according to the manufacturer’s instructions.

### 2.10 Western Blotting

Protein expression in CRBC cells cultured in 2D and 3D microbeads was analyzed by Western blot. Cells were lysed in RIPA buffer containing protease inhibitors on ice. Lysates were centrifuged at 12,000 rpm for 20 min at 4°C, and the supernatant was collected. Protein concentrations were determined using a BCA protein assay kit (P0012, Beyotime Biotech Inc., Shanghai, China). Equal amounts of protein (30 μg per sample) were separated on 10% SDS-PAGE gels and transferred to activated polyvinylidene fluoride (PVDF) membranes. After blocking with 5% skim milk, membranes were washed with TBST and incubated overnight with primary antibodies (anti-GAPDH, anti-β-Actin, anti-NANOG anti-ALDH1A1, and anti-SOX-2) targeting the proteins of interest. The next day, membranes were incubated with goat anti-rabbit IgG conjugated to horseradish peroxidase (HRP) for 2 h. Protein bands were visualized using enhanced chemiluminescence (80196, Thermo Fisher, Waltham, MA, USA) and imaged with the ChemiDoc MP system (Bio-Rad, Hercules, CA, USA).

### 2.11 Transwell Migration Assay of CRBC Cells

CRBC cells from 2D cultures and dissociated CRBC-Alg-GelMA microbeads were incubated at 37°C for migration assays. Microbeads were dissociated using GelMA lysis buffer. Freshly irradiated Swiss-3T3-J2 mouse fibroblasts (2 × 10^4^) were seeded into the lower wells of 24-well transwell plates (Corning Costar, New York, NY, USA). Once adherent, the medium was replaced with CRBC culture medium to support migration. Transwell inserts with 8 μm pore-sized membranes were placed in the wells. A serum-free medium was added to the upper chamber, and 2.5 × 10^4^ CRBC cells were seeded per insert. Each lower chamber received 750 μL of CRBC culture medium. Plates were incubated at 37°C for 24–48 h. Following incubation, cells were fixed with 4% formaldehyde at room temperature, washed twice with PBS (0.01 M, pH 7.4), and stained with 750 μL of crystal violet for 20 min. After staining, wells were washed twice with PBS, and non-migrated cells on the upper surface of the membrane were gently removed using cotton swabs. Migrated cells on the lower membrane surface were visualized and counted under a light microscope (CKX53, Olympus, Tokyo, Japan).

### 2.12 Drug Sensitivity Screening of CRBC Cells

CRBC cells (5 × 10^3^ cells per well) and CRBC-Alg-GelMA microbeads (one bead per well) were seeded into standard 96-well plates and incubated for 5 days at 37°C with 5% CO_2_. Antineoplastic drugs at various concentrations (Table A2) were added to the wells, with PBS (0.01 M, pH 7.4) used as the control. Drug screening was conducted in modified culture medium lacking Y-27632 to avoid interference with proliferation during treatment. After 4 days of drug exposure, cell viability was assessed according to the manufacturer’s instructions. Results were normalized to the PBS control and expressed as a percentage viability. The half-maximal inhibitory concentration (IC_50_) values were calculated using GraphPad Prism version 9.0 (GraphPad Software Inc., La Jolla, CA, USA). The heatmap of LogIC_50_ value were generated by applying nonlinear regression of GraphPad Prism version 9.0. The values are depicted with a color gradient from white (low) to blue (high).

### 2.13 Statistical Analysis

All data are expressed as mean ± standard deviation (SD) from at least three independent biological replicates. Statistical significance was assessed using Student’s *t*-test for comparisons between two groups and one-way ANOVA for comparisons among multiple groups. *p*-values < 0.05 were considered statistically significant. Significance levels were denoted as *p* < 0.05 (*), *p* < 0.01 (**), *p* < 0.001 (***), and *p* < 0.0001(****).

## 3 Results

### 3.1 Establishment of Patient-Derived Breast Cancer Cell Models

Breast cancer tissues and surrounding normal tissues were obtained from eight female patients treated at The Sixth Affiliated Hospital of South China University of Technology (see Table A1 for clinical details). The inclusion of peptides with different molecular types enables comprehensive evaluation of drug responses. Tissues were collected post-surgery, immediately stored in primary tissue storage solution, and processed within 4 h to ensure high success rates in establishing patient-derived cell cultures.

Patient-derived cells were established from breast cancer tissues using the conditional reprogramming technique to enable personalized therapy studies (see Scheme 1). Tumor and adjacent tissues were enzymatically digested using collagenase and DNase. The resulting cells were co-cultured with irradiated Swiss-3T3-J2 fibroblasts. Under 2D conditions, patient-derived CRBC cells proliferated in clusters surrounding the feeder cells, with aggregation areas expanding over time. Growth factors released by the feeder layer supported CRBC cells’ proliferation in this co-culture system.

**Figure 2:**
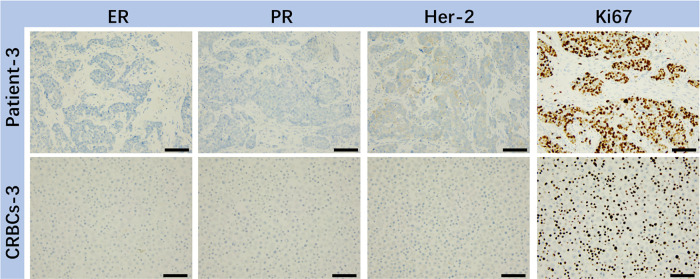
IHC analysis of various markers (ER, PR, HER-2, Ki67) on representative breast tumor sample from surgical patient and corresponding patient-derived CRBC cells. These images were taken from the microscope. The scale bars are 5 μm

To verify the genetic consistency between patient tumors and derived CRBC cells, immunohistochemical staining was performed (Fig. 2). CRBC cells from patient 3 (triple-negative breast cancer, TNBC) showed high Ki-67 expression and low ER, PR, and HER2 expression. These results confirm that CRBC cells retain key histological and molecular characteristics of their parental tumors, validating their use for downstream functional studies.

### 3.2 Construction and Stability of CRBC-Alg-GelMA Hydrogel Microspheres

**Figure A1:**
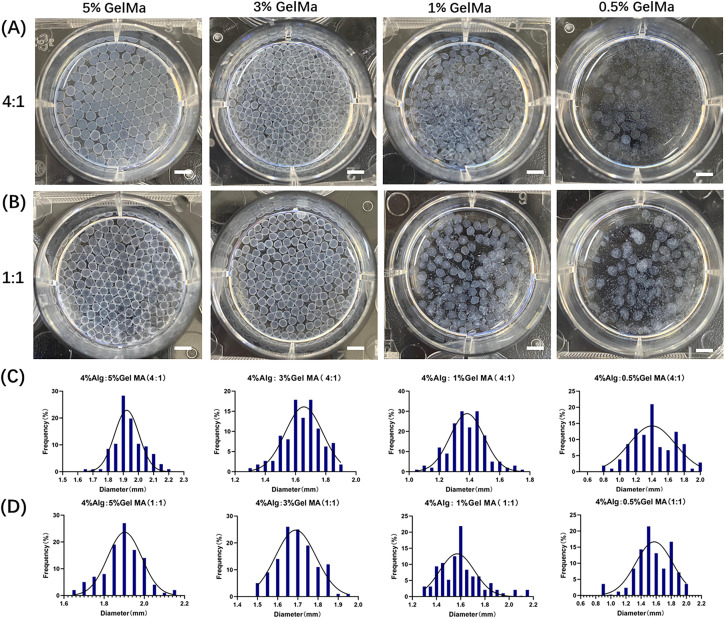
The characteristics of Alg-GelMA hydrogel microspheres. (**A**) The photos of microspheres were made using 4% alginate solution and gelatin solution with different concentrations (5%, 3%, 1%, 0.5%, from left to right) at a mixed ratio of 4:1. Scale bar, 0.5 mm. (**B**) The photos of microspheres were made using 4% alginate solution and gelatin solution in different concentrations (5%, 3%, 1%, 0.5%, from left to right) at a mixed ratio of 1:1. Scale bar, 0.5 mm. (**C**) The size distributions of corresponding samples of (**A**), respectively. (**D**) The size distributions of samples of (**B**), respectively

To validate the formation of the CRBC-Alg-GelMA microsphere model, we first evaluated the size and morphology of microspheres under different formulation conditions. As shown in Fig. A1, hydrogel microspheres formed well-defined, spherical structures when GelMA concentrations were 5% or 3%, and the alginate-to-GelMA ratios were 4:1 or 1:1. In contrast, at lower GelMA concentrations (1% and 0.5%), spherical integrity was lost. Among the tested conditions, the 4:1 alginate-to-GelMA ratio yielded microspheres with the most uniform, nearly spherical shapes and narrow size distributions when the GelMA concentration was 5% or 3%.

**Figure A2:**
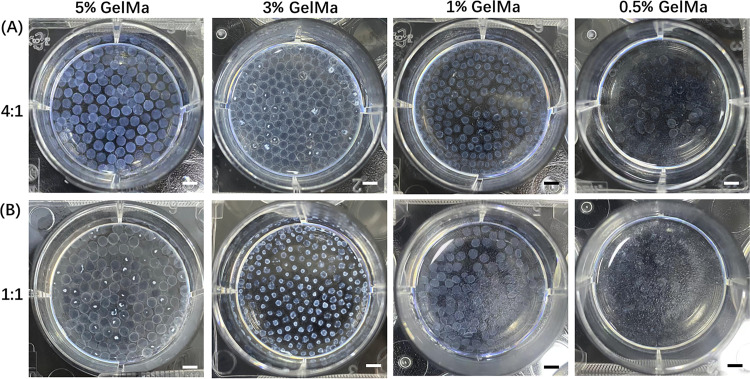
The changes of characteristics of Alg-GelMA hydrogel microspheres under different conditions. (**A**) The representative photos of Alg-GelMA hydrogel microspheres were made by 4% alginate solution and gelatin solution with different concentrations (5%, 3%, 1%, 0.5%, from left to right) at a mixed ratio of 4:1 after 5 days of storage. Scale bar, 0.5 mm. (**B**) The representative photos of Alg-GelMA hydrogel microspheres made by 4% alginate solution and gelatin solution with different concentrations (5%, 3%, 1%, 0.5%, from left to right) at a mixed ratio of 1:1 after 5 days of storage. Scale bar, 0.5 mm.

To assess stability, we examined the morphology and size of microspheres after 5 days of storage. As shown in Fig. A2, only microspheres formed with 4% alginate, 5% GelMA, and a 4:1 mixing ratio maintained their structural integrity, while those prepared under other conditions showed varying degrees of degradation. Therefore, we selected this formulation for all subsequent experiments. Using this optimized condition, we constructed individual CRBC-loaded hydrogel microspheres derived from patient breast cancer tissue. As illustrated in Scheme 1, CRBC cells were suspended in the Alg-GelMA solution (4% alginate, 5% GelMA, 4:1 ratio) and dispensed dropwise into 2% CaCl_2_ buffer using a 1 mL syringe. After 30 s of UV exposure, CRBC-Alg-GelMA hydrogel microspheres were successfully formed. The average diameter of the microspheres was 1.944 ± 0.308 mm, indicating good uniformity and reproducibility.

**Figure 3:**
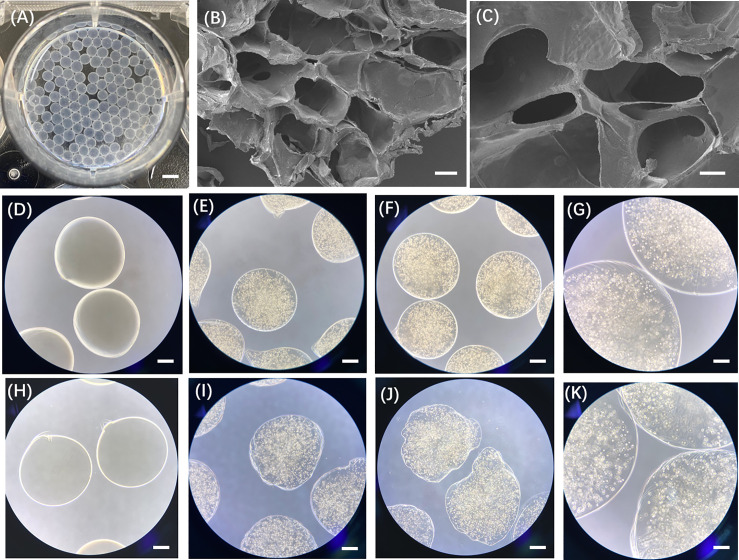
Characterization of patient-derived 3D CRBC-Alg-GelMA microbeads. (**A**) Photo of the prepared Alg-GelMA microbeads. Scale bar, 5 mm. (**B**,**C**) The internal morphology images of Alg-GelMA microbeads were captured using a scanning electron microscope (SEM). Scale bars are 100 μm and 40 μm, respectively. (**D**,**H**) The bright-field microscopy images of blank Alg-GelMA microbeads prepared at Alg/Gel mixing ratio of 4:1 and 1:1 (40× magnification). Scale bar, 0.5 mm. (**E**,**I**) The bright-field microscopy images of 3D CRBC-Alg-GelMA microbeads prepared at Alg/Gel mixing ratio of 4:1 and 1:1 (40× magnification). Scale bar, 0.5 mm. (**F**,**J**) The bright-field microscopy images of 3D CRBC-Alg-GelMA microbeads prepared at Alg/Gel mixing ratio of 4:1 and 1:1 (Alg:Gel) after 5 days storage (40× magnification). Scale bar, 0.5 mm. (**G**,**K**) The bright-field microscopy images of 3D CRBC-Alg-GelMA microbeads prepared at Alg/Gel mixing ratio of 4:1 and 1:1 (Alg:Gel) after 5 days storage (100× magnification). Scale bar, 0.2 mm

Field-emission scanning electron microscopy revealed that the microsphere surface possessed a porous structure with pore sizes ranging from 200 to 500 μm, which supports cell adhesion, growth, and interaction (Fig. 3). Compared to microspheres prepared at a 1:1 alginate-to-GelMA ratio, those prepared at a 4:1 ratio demonstrated superior stability after 5 days of storage. Therefore, the CRBC-Alg-GelMA microspheres produced under these conditions were used in subsequent experiments.

### 3.3 Growth and Stability of CRBC-Alg-GelMA Hydrogel Microspheres

**Figure 4:**
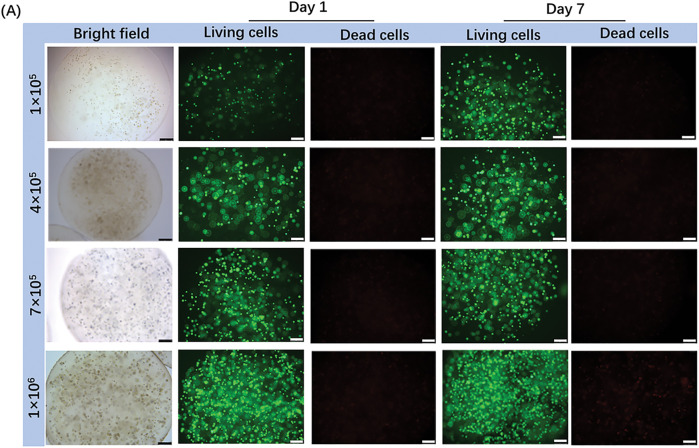

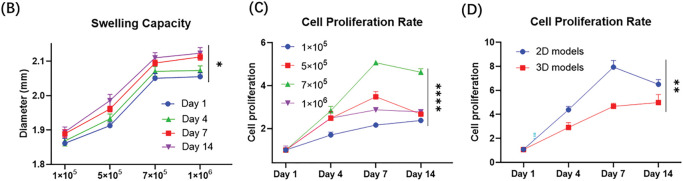
The growth and stability of patient-derived 3D CRBC cells microbead models. (**A**) The bright field and fluorescence images of 3D CRBC cells microbead models after seeding different numbers of cells (1 × 10^5^, 5 × 10^5^, 7 × 10^5^, 1 × 10^6^, respectively) after 1 day and 7 days. Green fluorescence indicates live cells and red fluorescence indicates dead cells; scale bars are 250 μm. (**B**) Anti-swelling performance analysis of 3D CRBC microbead models in different numbers of seeding cells after culturing for 1, 4, 7, and 14 days. (**C**) The cell proliferation curves of 3D CRBC microbead models in different numbers of seeding cells. (**D**) The cell proliferation curves of 2D culture models and 3D CRBC cells microbead models over 14 days. The data are presented as mean ± SD (n = 3). *p < 0.05, **p < 0.01, ****p < 0.0001

**Figure A3:**
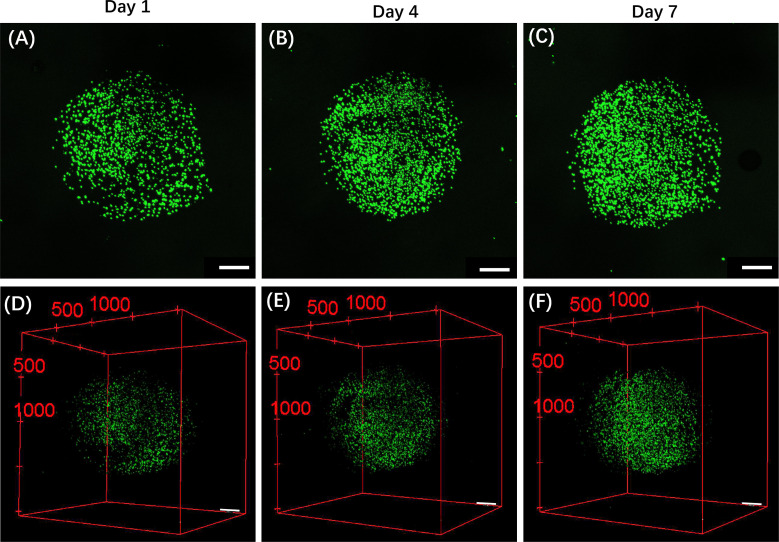
The fluorescence composite images of CRBC cell microbead models during the growth process. (**A**,**B**,**C**) The fluorescence z-stacked composite images of CRBC cell microbead models after seeding 1, 4 and 7 days. The scale bars are 500 μm. (**D**,**E**,**F**) The 3D z-stack reconstruction of fluorescence images of CRBCs microbead models after cell seeding for 1, 4 and 7 days. Green fluorescence indicates the live cells. The scale bars are 500 μm

Live/dead staining was used to assess the viability of patient-derived CRBC cells encapsulated in Alg-GelMA microspheres. As shown in Fig. 4, fluorescence imaging revealed a notable increase in live cell numbers after 7 days of culture, regardless of the initial cell seeding density. The growth of cells in 3D models was detected by the confocal fluorescence imaging with z-stack reconstructions (Fig. A3). The results showed that the cells grew in the three directions of the sphere models. Microsphere diameters were measured on days 1, 4, 7, and 14. A slight increase in size was observed during the first 7 days, after which the microspheres maintained stable dimensions, indicating good anti-swelling properties during extended culture. Proliferation rates were dependent on initial cell density: lower cell concentrations resulted in slower growth, while higher densities promoted faster proliferation. However, proliferation reached a plateau around day 7 and declined thereafter. These findings suggest that optimal culture conditions include limiting cell numbers to approximately 7 × 10^5^ and maintaining the culture duration under 7 days. Compared to 2D models, the 3D CRBC-Alg-GelMA microsphere system exhibited a more gradual growth curve, better reflecting *in vivo* tumor behavior and offering a more physiologically relevant model for preclinical drug screening.

### 3.4 Phenotypic Characteristics of CRBC in 2D vs. 3D Culture Models

**Figure 5:**
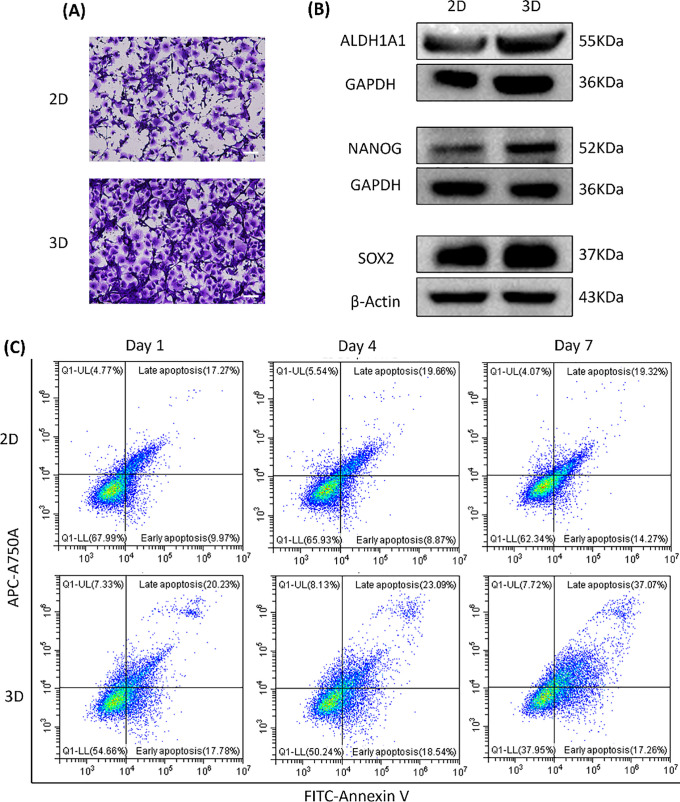
The differences between patient-derived 2D culture models and 3D CRBC cell microbead culture models. (**A**) Cell migration of 2D monolayer models and 3D CRBC cell microbead models. The scale bars are 100 μm. (**B**) Protein expression levels of general stem cell markers (ALDH1A1, NANOG, SOX-2) of 2D monolayer and 3D CRBC microbeads models after culturing. (**C**) Cell apoptosis of 2D monolayer models and 3D CRBC microbead models was measured by a flow cytometer on the 1, 4, and 7 days of culture

To compare tumor cell behavior under different culture conditions, we examined phenotypic differences between 2D monolayers and 3D CRBC-Alg-GelMA microsphere models. As shown in Fig. 5A, CRBC cells cultured in 3D exhibited an enhanced migratory ability compared to those in 2D cultures, suggesting superior growth and metastatic potential. These differences may be attributed to the tumor microenvironment (TME), which is known to support tumor stemness.

Cancer stem cell traits are closely linked to tumor progression, recurrence, and treatment resistance [25]. Studies indicate that 3D cultures better mimic the tumor microenvironment (TME) and enhance cancer stem cell maintenance, partly through the upregulation of stemness-associated transcription factors such as SOX-2 and NANOG [26]. We analyzed the expression of stemness-related markers ALDH1A1, NANOG, and SOX-2 in both models (Fig. 5B). The 3D-cultured CRBC cells showed significantly higher expression of these proteins compared to cells in 2D cultures.

Cell apoptosis was assessed using FITC/PI staining and flow cytometry (Fig. 5C). The proportion of apoptotic cells (in both early and late stages) was higher in the 3D model than in the 2D culture, possibly due to enhanced apoptosis signaling induced by the 3D microenvironment. This response may better reflect the apoptotic profiles seen in patient tissues [27]. Overall, the 3D CRBC-Alg-GelMA microsphere model more accurately replicates *in vivo* conditions, maintaining tumor-like morphology and microenvironmental cues. It promotes tumor stemness and preserves proliferative capacity, making it a more physiologically relevant platform for drug screening compared to conventional 2D models.

### 3.5 Drug Screening Conditions of 3D CRBC-Alg-GelMA Microsphere Models

**Figure A4:**
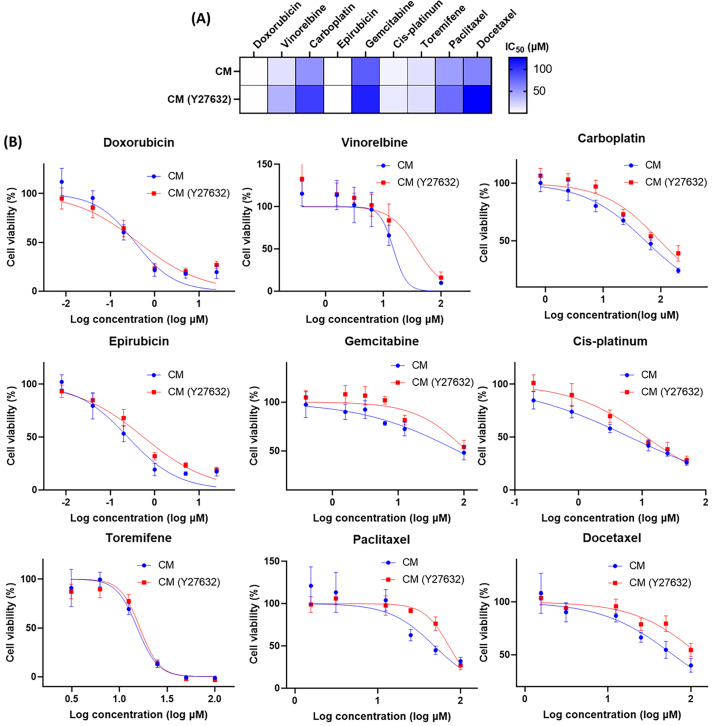
The evaluation of pharmaceutical effects under different culture conditions (CM or CM with Y-27632). (**A**) Heatmap of the IC_50_ values for the drugs in Y-27632 added complete medium (CM) and CM by applying nonlinear regression. (**B**) Dose-response curves for the medications in the 3D culture models. All data is presented as mean ± SD (n = 3)

**Figure A5:**
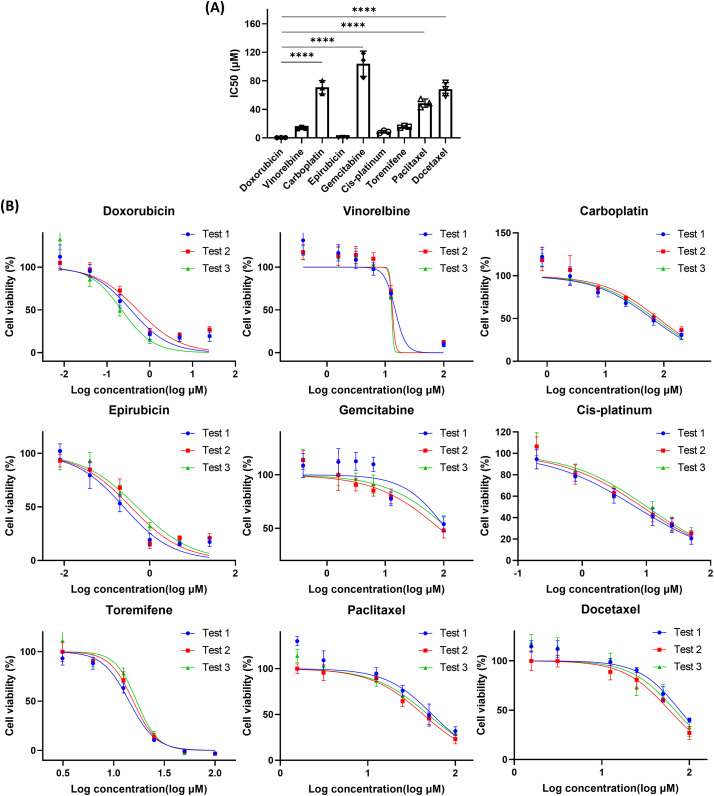
The cell viability of 3D CRBC culture models after various drug treatments. (**A**) The IC_50_ values of different drugs on 3D culture models from patient-derived CRBC cells. The data are presented as mean ± SD (n = 3). ****p < 0.0001. (**B**) Dose-response curves of various medicines on 3D culture models of different passages of CRBC cells. All data is presented as mean ± SD (n = 3)

The ROCK inhibitor Y-27632, commonly used in conditional reprogramming culture, enhances the proliferation of primary cells by inhibiting Rho-associated kinase activity [28]. However, its impact on drug response remains unclear. To investigate this, we prepared drug dilutions in complete medium with or without Y-27632 and evaluated the IC_50_ values of various anti-cancer drugs in 3D CRBC-Alg-GelMA microsphere models (Fig. A4). The results showed that Y-27632 increased the IC_50_ values of several drugs, including vinorelbine, carboplatin, gemcitabine, and docetaxel, suggesting it may attenuate drug sensitivity. Previous studies have shown that short-term withdrawal of Y-27632 does not significantly affect CRBC cells’ proliferation or induce senescence [29]. Therefore, to ensure accurate drug response assessment, Y-27632 should be excluded from the culture medium during drug sensitivity testing. To further validate the utility and reproducibility of the 3D CRBC-Alg-GelMA microsphere platform, drug screening was conducted across different cell passages. Microspheres were prepared using CRBC cells at various passages and exposed to drugs at gradient concentrations for 4 days (Fig. A5). The relative cell viabilities were consistent across passages, indicating that the model supports reliable and reproducible drug screening. These results demonstrate that the 3D CRBC-Alg-GelMA microsphere model is a robust platform for evaluating patient-specific drug responses and is suitable for large-scale screening applications.

### 3.6 Personalized Drug Evaluation Using Patient-Derived 3D Culture Models

**Figure 6:**
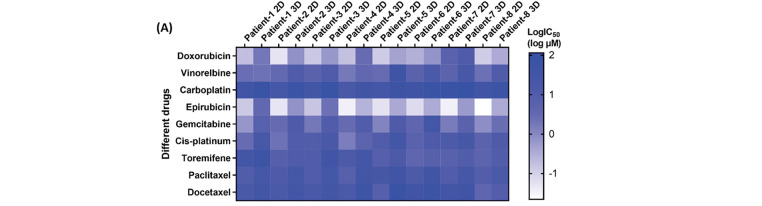

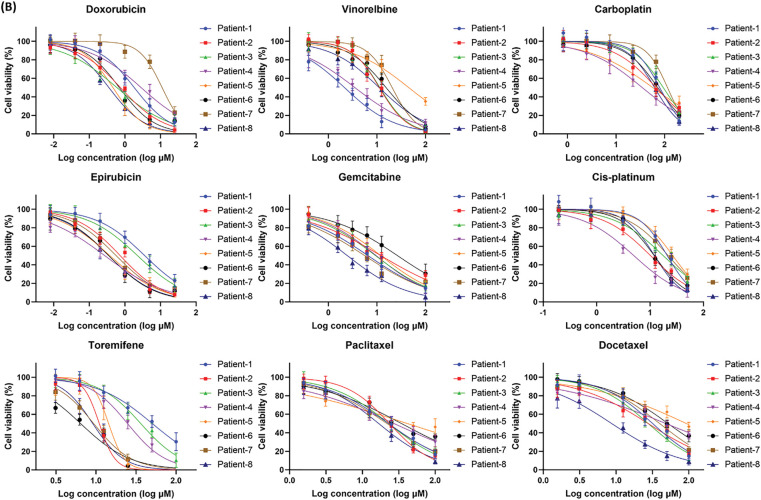
Anti-tumor drug evaluation of the CRBC cells under the conditions of 2D and 3D culture. (**A**) Heatmap of the LogIC_50_ values for the drugs cultured under the conditions of 2D and 3D models. (**B**) Dose-response curves for the different medicines of the patient-derived 3D models. All data is presented as mean ± SD (n = 3)

Drug sensitivity of patient-derived CRBC cells was assessed in both 2D and 3D culture models using cell viability assays. Cells were treated with various drugs at gradient concentrations for 4 days. A heatmap of IC_50_ values (Fig. 6A) revealed that CRBC cells in 3D cultures generally exhibited higher IC_50_ values than those in 2D cultures, suggesting increased drug resistance under 3D conditions. This may be attributed to enhanced cancer stemness and a more physiologically relevant tumor microenvironment in the 3D models. Thus, IC_50_ values derived from 3D cultures may more accurately reflect clinical drug responses. Dose-response curves from different patient-derived 3D models (Fig. 6B) showed patient-specific variations in drug sensitivity. For example, 3D models from patient 1 and patient 4 were more responsive to vinorelbine, while models from patient 6 and patient 7 responded more strongly to toremifene. The most effective drug for patient 7 was epirubicin, whereas doxorubicin was most effective for patient 8. These results underscore the potential of patient-specific 3D CRBC models to guide personalized therapeutic strategies in breast cancer treatment.

## 4 Discussion

With growing insights into the molecular mechanisms of breast cancer, targeted therapies have been developed to modulate key proteins, leading to improved survival and treatment outcomes. However, significant challenges remain in clinical practice [30]. Current molecular classifications do not fully capture the heterogeneity of breast cancer, limiting their utility in guiding therapy. While some patients with specific mutations respond well to targeted treatments, others, particularly those with advanced disease, show limited or incomplete responses. Therefore, *in vitro* methods to predict and screen individual patients’ sensitivity to anti-cancer drugs are essential for improving the effectiveness of personalized therapies.

Traditional drug screening strategies typically use immortalized tumor cell lines cultured in flat plates and exposed to drugs at varying concentrations to assess treatment efficacy based on cell viability [31]. However, this approach has several limitations [32]. First, source limitation: immortalized cells only partially represent the biology of clinical tumors. Interpatient variability in gene expression and epigenetic regulation significantly impacts drug response, underscoring the need for individualized data on drug sensitivity to support precision medicine. Second, plate culture limitation: conventional 2D culture methods fail to replicate the complexity of the *in vivo* tumor microenvironment, which includes not only tumor cells but also immune and stromal cells. Interactions between cells and the extracellular matrix play a critical role in modulating therapeutic responses. Therefore, developing 3D culture systems or organoid models that more accurately mimic the *in vivo* tumor context offers a more reliable strategy for evaluating tumor drug sensitivity *in vitro*. Whereas, matrix gel and tissue spheres which are commonly used in many works, still have some restrictions. Hydrogel has more advantages in terms of biocompatibility, tunability of physical and chemical properties, mechanical support, material transfer efficiency, as well as low cost, and becomes a promising object of study.

In this study, we used conditional reprogramming technology to isolate tumor cells from patient tissues and optimized conditions for constructing 3D hydrogel microspheres to establish CRBC-based 3D culture models for anti-cancer drug screening. CRBC cells cultured in 3D maintained continuous proliferation and formed distinct cellular clusters. Compared to 2D cultures, cells in the 3D system exhibited enhanced migration, spheroid formation, and elevated expression of stemness-related genes. Furthermore, the 3D models displayed greater drug resistance than 2D cultures, better reflecting *in vivo* tumor behavior. Similar findings have been reported in other disease models. Yau et al. demonstrated that 3D colorectal cancer (CRC) spheroids more accurately recapitulate tumor pathophysiology and show increased chemoresistance marker expression compared to 2D cultures [33]. Ren et al. demonstrated that patient-derived organoids of biliary tract cancer (BTC) exhibited variable responses to chemotherapies, including gemcitabine, cisplatin, 5-fluorouracil, and oxaliplatin [34]. Kim et al. developed a 3D rheumatoid arthritis (RA) model using macroscale-patterned polycaprolactone (PCL) scaffolds, enabling personalized assessment of immune interactions and drug responsiveness [35]. These findings support that increased drug resistance in 3D models more closely mirrors clinical scenarios, likely due to enhanced cell stemness and tumor microenvironmental influences.

Nevertheless, this study still has several limitations. First, the prepared patient-derived 3D organoids did not incorporate the tumor microenvironment construction, including the vascular structures and immune cells, etc. The *in vitro* remodeling of patient’s immune microenvironment is the obstruction to precisely predict clinical immunotherapy and targeted therapy. Besides, another limitation is the patients’ sample size. The establishment of patient-derived breast cancer organoid library can be served as a patients’ genetic platform for predicting drug sensitivity, choosing effective drugs, and further conducting mechanism research and validation in the large patient cohorts. In the future, we plan to incorporate additional factors such as multi-drug combinations and co-culture with various cell types in 3D models to better assess drug sensitivity. We aim to develop verification systems that more closely mimic clinical conditions, including vascularized and multicellular 3D platforms. These advances will enable more accurate evaluation of drug resistance and therapeutic indications, ultimately supporting precision medicine in clinical treatment.

## 5 Conclusion

This study demonstrated the advantages of Alg-GelMA hydrogel microspheres as a 3D cell culture platform. Using conditional reprogramming technology, we cultivated cells from surgical breast cancer patients and combined them with 3D hydrogel microspheres to establish patient-derived 3D culture models. Compared to 2D cultures, these 3D models exhibited higher expression of stemness genes and enhanced migratory capacity. We successfully developed eight patient-derived 3D CRBC models and screened them against nine anti-cancer drugs to evaluate individualized drug sensitivities. These models effectively reflected patient-specific drug responses, offering a valuable tool for clinical drug screening and addressing drug resistance. In summary, our patient-derived 3D CRBC-Alg-GelMA microsphere culture models represent a promising platform for precision medicine in breast cancer therapy.

## Data Availability

The data analyzed in this study are available from the corresponding author upon reasonable request.

## Appendix A

Development of patient-derived conditionally reprogrammed 3D breast cancer culture models for drug sensitivity evaluation .

**Table A1:** Clinical characteristics and sample collection information of breast cancer cohort

Case	Sex	Age (years)	Primary	Histological type	TNM stage	Molecular subtype	Immunohisto chemistry
Patient-1	Female	65	Primary	IDC*	IIB	Luminal B2	ER+, PR−, HER-2+, Ki67+
Patient-2	Female	48	Primary	IDC	IIB	Luminal A	ER+, PR+, HER-2+,Ki67+
Patient-3	Female	61	Primary	IDC	IIIA	TNBC	ER−, PR−, HER-2−, Ki67+
Patient-4	Female	47	Primary	IDC	IIB	HER-2+	ER−, PR−, HER-2+, Ki67+
Patient-5	Female	52	Primary	IDC	I	Luminal B1	ER+, PR+, HER-2−, Ki67+
Patient-6	Female	43	Primary	IDC	IIA	Luminal B1	ER+, PR+, HER-2−, Ki67+
Patient-7	Female	52	Primary	IDC	IIA	Luminal B1	ER+, PR+, HER-2−, Ki67+
Patient-8	Female	48	Primary	IDC	IIA	Luminal A	ER+, PR+, HER-2+, Ki67+

Note: *IDC, invasive ductal carcinoma; TNBC, triple-negative breast cancer; HER-2, human epidermal growth factor receptor 2; ER, estrogen receptor; PR, progesterone receptor; +, positive expression; −, negative expression.

**Table A2:** The concentrations of antineoplastic drugs during drug screening

Name	Concentration					
Carboplatin	5 mM	200 μM	66.67 μM	22.22 μM	7.41 μM	2.47 μM
Cis-platinum	1 mM	50 μM	25 μM	12.5 μM	3.125 μM	0.781 μM
Paclitaxel	10 mM	100 μM	50 μM	25 μM	12.5 μM	3.125 μM
Docetaxel	10 mM	100 μM	50 μM	25 μM	12.5 μM	3.125 μM
Gemcitabine	10 mM	100 μM	12.5 μM	6.25 μM	3.125 μM	1.563 μM
Vinorelbine	5 mM	100 μM	12.5 μM	6.25 μM	3.125 μM	1.563 μM
Doxorubicin	10 mM	25 μM	5 μM	1 μM	0.2 μM	0.04 μM
Epirubicin	5 mM	25 μM	5 μM	1 μM	0.2 μM	0.04 μM
Toremifene	10 mM	100 μM	50 μM	25 μM	12.5 μM	6.25 μM
